# Rhinitis, sinusitis and ocular disease – 2103. Comparative allergen profile in Krishna – Godavari region

**DOI:** 10.1186/1939-4551-6-S1-P177

**Published:** 2013-04-23

**Authors:** Hari Kishan Gonuguntla, Bhanu Naik Bhukya, Prabhakar Rao Parankusam

**Affiliations:** 1 Pulmonology, Katuri Medical College & Hospital, Guntur, India

## Background

Krishna(K) and Godavari(G) regions of Andhra Pradesh have high prevalence of allergic diseases due to increasing number of paper mills, stone quarrying, tobacco crops and chilli plantations.

## Aims and objectives

Primary objective is to determine the prevalence of major allergens in these areas. A Secondary objective is to draw a relation between asthma and allergy in these patient groups.

## Materials and methods

Allergy testing in susceptible individuals was done by skin prick test. A total of 100 patients (n=100, G=50, K =50) were included in the study.Detailed history of the patient was obtained by using a model questionnaire. Patients were abstained from taking any antihistamines, antipsychotics, one week prior to the date of procedure. Skin prick test was done using antigen solutions (total 150 antigens) for pollens,insects &dust. A relationship was drawn on the major allergens prevalent in this area, association of allergic rhinitis & asthma from the test results.

## Results

Among the 100 patients, skin prick test was positive in 80(K= 36, G =44) patients for at least one antigen. Of these allergic rhinitis was confirmed in 30 patients(K=13,G=17), Bronchial asthma in 21 patients(K=11,G=10), Rhinitis +Bronchial asthma in 11 patients(K=6,G=5), and 20 patients(K=20,G=18) had no history of either asthma or rhinitis based on their history. House dust mite was found to be the common major allergen prevalent in both regions. Among pollens Adathoda, Azeratum , Azadirachta, Cenchrus , Gynandropsis were more prevalent in Godavari region while Brassica, Albizia , Azadirachta, Cenchrus , Chenopodium were prevalent in Krishna region. Among insects mosquito allergy was predominant among Krishna region while cockroack allergy was more in Godavari region. Among dust, Paper dust allergy(57% in G vs. 8% in K region) is more in Godavari while Grain dust was more in Krishna region.

## Conclusion

1. House dust mite is the major allergen in both regions.

2. Pollen allergy is highly prevalant in these regions major being Brassica & Adathoda.

3. Paper dust allergy is more in Godavari compared to Krishna due to high prevalence of paper mills in that region.

4. Mosquito allergy is more among Krishna region as it is an area known for stagnant water.

